# Bacterial lipopolysaccharide forms aggregates with apolipoproteins in male and female rat brains after ethanol binges

**DOI:** 10.1016/j.jlr.2024.100509

**Published:** 2024-01-29

**Authors:** L. López-Valencia, M. Moya, B. Escudero, B. García-Bueno, L. Orio

**Affiliations:** 1 Department of Psychobiology and Behavioral Sciences Methods, Faculty of Psychology, Complutense University of Madrid, Pozuelo de Alarcón, Spain; 2 Instituto de Investigación Sanitaria Hospital Universitario 12 de Octubre (imas12), Neuroscience and Mental Health, Madrid, Spain; 3 Department of Pharmacology and Toxicology, Faculty of Medicine, Complutense University of Madrid (UCM), Neurochemistry Research Institute UCM (IUIN), Madrid, Spain; 4 Biomedical Network Research Center of Mental Health (CIBERSAM), Institute of Health Carlos III, Madrid, Spain; 5 RIAPAd: Research network in primary care in addictions (‘Red de investigación en atención primaria en adicciones’), Spain

**Keywords:** alcohol, binge drinking, LPS, Lipid A, apolipoprotein, TLR4, ApoAI, ApoB, neuroinflammation, sex-differences

## Abstract

Alcohol binge drinking allows the translocation of bacterial lipopolysaccharide (LPS) from the gut to the blood, which activates the peripheral immune system with consequences in neuroinflammation. A possible access/direct signaling of LPS to/in the brain has not yet been described under alcohol abuse conditions. Apolipoproteins are compounds altered by alcohol with high affinity to LPS which may be involved in its transport to the brain or in its elimination. Here, we explored the expression of small components of LPS, in its free form or bound to apolipoproteins, in the brain of female and male rats exposed to alcohol binges. Animals received ethanol oral gavages (3 g/kg every 8 h) for 4 days. LPS or its components (Lipid A and core), LPS-binding protein, corticosterone, lipoproteins (HDL, LDL), apolipoproteins (ApoAI, ApoB, and ApoE), and their receptors were measured in plasma and/or in nonperfused prefrontal cortex (PFC) and cerebellum. Brain LipidA-apolipoprotein aggregates were determined by Western blotting and confirmed by co-immunoprecipitation. In animals exposed to alcohol binges: 1) plasma LPS-binding protein was elevated in both sexes; 2) females showed elevations in plasma ApoAI and corticosterone levels; 3) Lipid A formed aggregates with ApoAI in the female PFC and with ApoB in males, the latter showing Toll-like receptor 4 upregulation in PFC but not females. These results suggest that small bacterial components are present within the brain, forming aggregates with different apolipoproteins, depending on the sex, after alcohol binge intoxications. Results may have implications for the crosstalk between alcohol, LPS, and neuroinflammation.

## Graphical abstract

**Figure:**
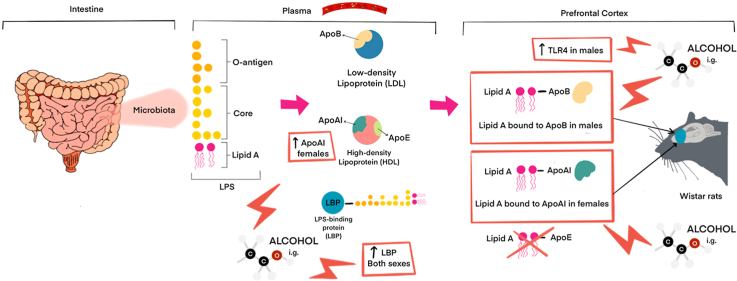

Alcohol binge drinking (ABD) is one of the most widespread patterns of alcohol consumption worldwide among adolescent people. It consists of a heavy intake of alcohol that brings blood ethanol concentration (BEC) to ≥ 0.08 g/dl in a short period of time. It means five or more alcoholic beverages for males and four or more in females in approximately two hours (1, 2). This consumption produces acute and chronic effects, which may affect organs such as the brain, gut, and liver. ABD affects the structure and/or function of the central nervous system (CNS), including loss of white matter and behavioral alterations such as cognitive impairment. Anyone can be susceptible to its deleterious effects, especially adolescents, due to their immature prefrontal cortex (PFC), which is responsible for executive functions and impulse control (3, 4, 5). Many of these changes during adolescence may remain into adulthood in rat models (6).

Other organs also affected by ABD are the gut and the liver. ABD induces gut dysbiosis, promoting an increase in *Gram*-negative bacteria, gut inflammation, and disruption of the intestinal barrier, allowing the translocation of bacteria to the mesenteric lymph nodes and bacterial components, such as lipopolysaccharide (LPS), to the systemic circulation, an effect known as leaky gut (7, 8, 9, 10, 11).

These events have been proposed as possible triggers of the well-known peripheral inflammatory and neuroinflammatory response induced by alcohol, activating the innate immune system by increasing the TLR4 receptor, inducing the translocation of the nuclear factor *kappa* B transcription factor to the nucleus, and promoting the expression of pro-inflammatory mediators such as cytokines, chemokines, and high-mobility-group box protein 1, which are related to cellular damage (6, 12, 13). Activation of the TLR4 proinflammatory pathway has been directly related to some behavioral disturbances during abstinence, such as depressive-like behavior, anxiety, or anhedonia (14, 15).

As mentioned above, LPS is a large component of the outer membrane of *Gram*-negative bacteria (16). It consists of three different parts attached to each other: Lipid A, a glycolipid domain that determines toxicity and inflammation mainly by activating TLR4; the core, a short chain of sugar residues; and the O-antigen, a highly variable polysaccharide moiety (17, 18). Under physiological conditions, low circulatory levels of LPS have been detected in the plasma of animals (19) and humans (17). LPS-induced cellular responses are the net result of the interaction of LPS with various plasma components, such as soluble cluster of differentiation 14 (CD14), LPS-binding protein (LBP), phospholipid transfer protein, and membrane receptors, such as membrane-bound CD14 and TLR4. Balanced cellular responses are essential for the host defense against bacterial infections. However, if large amounts of LPS are present in the circulation, an excessive cellular response can be deleterious for the host, and therefore, endotoxin-inactivating processes are of extreme importance (18). There are several routes of LPS detoxification in the circulation, but the most relevant is by its incorporation into lipoproteins (20).

Lipoproteins can be classified according to their density as very low-density lipoprotein (VLDL), low-density lipoprotein (LDL), and high-density lipoprotein (HDL). Their protein part is composed of apolipoproteins (Apos) (type AI or E in HDL (20), type B in LDL (21), and type B100 or E in VLDL) (22). Apolipoproteins transport lipids in polar water-based solutions such as blood, cerebrospinal fluid, and lymph. Apolipoproteins interact with membrane lipoprotein receptors and lipid transfer proteins to regulate lipoprotein uptake and clearance and serve as enzyme cofactors for enzymes implicated in the metabolism of lipoproteins (23). There is increasing evidence supporting the idea that apolipoproteins are involved in functions beyond cholesterol transport (24), such as the detoxification of bacterial LPS as actors of the innate immune system (25). The first step in LPS detoxification is its binding to HDL. Once LPS is bound to HDL, its inflammatory activity is reduced. Although HDL may protect initially, LPS should be moved to LDL because the `buffering' capacity of HDL is quickly exceeded (26). In a second step, acute phase transfer proteins LBP and phospholipid transfer protein efficiently redistribute LPS from HDL to other lipoprotein subclasses, such as LDL and VLDL, in a time-dependent manner (27). Pathogen lipids incorporated into LDL and VLDL are then cleared by the liver via the LDL receptor (LDLr) (and possibly other lipoprotein receptors such as VLDL receptor, ApoE receptor 2 (ApoER2), and scavenger receptor class B type 1 (SR-BI)) and secreted in bile (19).

One controversial question is how peripheral LPS can stimulate the activation of the innate immune response in the brain (28). Structures lacking the blood brain barrier (BBB), such as the circumventricular organs, and other blood‒brain interfaces, such as the choroid plexus and the meninges, rapidly respond to pro-inflammatory stimuli by LPS (29). The direct infiltration of LPS into brain tissue has been questioned (30), but several, not exclusive between them, humoral/cellular routes have been proposed: 1) by means of cytokine signaling through the vagus nerve; 2) increased BBB permeability; 3) vascular prostaglandin effects; 4) leukocyte infiltration. We have recently shown that small components of *Escherichia Coli* LPS (Lipid A and Core) may infiltrate the brain under physiological conditions bound to apolipoproteins (31). Specifically, we observed LipidA-apolipoprotein aggregates in tanycytes-like cells (interface between blood and CSF) and ependymal cells in circumventricular organs, but we also observed positive staining in brain-endothelial cells, for example, in the hippocampal commissure, and even in astrocytes of the medulla oblongata (31). Astrocytes and tanycytes in the circumventricular organs of the brain are known to be crucial for initiating the LPS-induced inflammatory responses via TLR4 (32). Interestingly, we also observed minimal presence of Lipid A aggregates in the PFC of perfused animals under physiological conditions.

It is not clear at present whether the binding of LPS to apolipoproteins is involved in the transport or signaling of LPS to the brain or it is a mechanism for endotoxin detoxification. The first would indicate a direct mechanism for LPS signaling in the brain, with consequences in neuroinflammation; the latest would indicate an attempt of the body to neutralize alcohol-induced neuroinflammation by forming LPS-apolipoprotein aggregates. Whatever the functional consequences, an interesting preliminary research question is to investigate the state of LPS components and apolipoproteins in the brain of animals exposed to alcohol intoxications, since alcohol abuse disrupts the BBB and induces neuroinflammation (33).

In the present study, we aimed to explore whether there are differences in the expression of small LPS components, apolipoproteins, or their aggregates in animals exposed to alcohol binge intoxications compared with controls. We explored two brain structures affected by alcohol, such as the PFC and the cerebellum.

We hypothesized that H1) animals which undergo alcohol binge intoxications show more expression of small LPS components (Lipid A or Core) or their aggregates with apolipoproteins in the brain than control animals; H2) H1 could differ in male and female animals exposed to alcohol binge intoxications and in different brain regions.

## Materials and Methods

### Animals

Forty-four Wistar rats (Envigo©, Barcelona, Spain) aged six weeks were used across all experiments. Upon arrival, females weighed 160–220 g and males 180–230 g and were housed in different isolated rooms. Animals were housed in groups of 2–3 per cage and maintained at constant conditions of temperature (21 ± 1°C) and humidity (59 ± 10%) under a 12 h dark-light inversed cycle (lights on at 8:00 p.m.) with free access to food and water. Animals were habituated to these conditions for 11 days before the experiments, at which time they were handled gently to acclimate to the experimenters and gavage procedure.

All procedures were approved and adhered to the guidelines of the Animal Welfare Committee of the Complutense University of Madrid (Ethical approval reference: PROEX 312/19) following European legislation (2010/63/EU).

### Experimental design and ethanol intoxication procedure

Animals were randomly assigned to control and experimental groups: male control group, male ethanol group, female control group, and female ethanol group. Rats received intragastric (i.g.) ethanol or water three times per day using specific cannulae (16-G needle, Fisher Scientific, Waltham, MA), following a standard paradigm of a 4-days binge alcohol intoxication protocol previously used by our group (10, 15) and by others (34). An additional control group of animals was used in a pilot study to compare the effects in saline perfused versus nonperfused animals prior brain extraction.

Ethanol solutions were prepared daily from 96% ethanol stock diluted in water, and body weights were measured daily 120 min before the beginning of the i.g. gavage. Female and male ethanol-treated rats received an initial loading dose of 5 g/kg in a 30% solution (w/v) and then a maximum of 3 g/kg for additional doses (Supplemental Table S1). This repeated binge-pattern ethanol paradigm maintained relatively constant intoxicating BEC in a range of sedation/ataxia according to the 6-point behavioral ethanol intoxication scale (35). In this study, the average dose of ethanol per rat was 8.73 g/kg/day.

The female reproductive cycle was controlled during the experiment by collecting vaginal smears once a day at the same time of the day to reduce variability. Vaginal secretions were collected with a plastic pipette filled with normal saline (NaCl 0.9%) by introducing the tip gently into the rat vagina, and the vaginal fluid was placed on different glass slides and immediately examined under a light microscope. Estrous cycle phases were determined by observation of cell types in the entire smear (36) by using a Nikon Japan microscope (Nikon Instruments, Inc., Melville, NY) (Supplemental Fig. S1).

### Tissue and plasma collection

Following an alternation of the four experimental groups, samples were taken three hours after the last ethanol administration prior to administration of a lethal dose of sodium pentobarbital (320 mg/kg, i.p., Dolethal®, Spain). Blood was collected by cardiac puncture using ethylenediaminetetraacetic acid (molecular weight 452.24 g/mol, pH 7.2) as an anticoagulant, and then animals were decapitated. Blood samples were centrifuged at 4°C for 15 min at 2,000 g for plasma fraction collection, which was stored at −80°C until assay. Brains were rapidly isolated from the skull, discarding blood vessels and meninges, and the PFC was excised and frozen at −80°C until assayed.

Using this experimental protocol for samples collection, we reproduce real conditions as much as possible, since the blood-flow in the brain vessels could be an important source of molecules that drive the alcohol-induced neuroimmune response. In this sense, it has been proposed that circulating factors greatly retard the interaction of LPS with the BBB, and the luminal binding of LPS is enhanced when those factors are removed (30). Although washing the vascular space of the brain does not reproduce real conditions, we have used this complementary experimental approach in a pilot study using an independent group of animals that were intracardially perfused with sterile physiological saline solution (0.9% NaCl) prior brain collection to check the contribution of PFC blood flow in the observed effects.

### Western blot analysis

Brain samples were homogenized by sonication in PBS (pH = 7.4) mixed with a protease inhibitor cocktail (Complete, Roche®, Madrid, Spain) at a dilution of 1:3 (w/v), followed by centrifugation at 13,000 rpm at 4°C for 10 min. Protein levels were measured and adjusted by Bradford’s method, and homogenates were mixed with Laemmli simple buffer (Biorad®, Alcobendas, Madrid, Spain) containing β-mercaptoethanol (50 μl/ml of Laemmli) to obtain a final concentration of 1 mg/ml. Proteins were separated by an electrophoresis gel, blotted onto nitro-cellulose membranes (Amersham Ibérica®, Madrid, Spain) with a semidry transfer system (Bio-Rad®, Madrid, Spain), incubated with specific primary and secondary antibodies (Supplemental Table S2), and revealed by using a chemiluminescence system (ECL™-kit) (Amersham Ibérica®, Madrid, Spain). Autoradiographs were quantified by densitometry (NIH ImageJ® software, National Biosciences, Lincoln, Nebraska) and expressed as optical density (O.D.). In all Western blot analyses, the housekeeping β-actin protein was used as a loading control. Every blot contained different samples per group, and two blots were run in separate assays. The results represent the average of two technical replicates.

To maximize the analysis of multiple proteins with a limited tissue, the membranes in western blots were cut, and each small membrane was incubated with the antibody of interest. The blots are represented in the images as they were loaded (vertical distribution before cutting the membrane). When necessary, a stripping procedure was performed, as indicated in the representative blots of the figures. The samples of Western blot were loaded separately for male and female groups, so no direct comparation between sexes was done in these analyses.

In a pilot study, we demonstrate that Lipid A is bound to different proteins, all of which are of interest in our study. This binding activity was visualized by both Western blot and coimmunoprecipitation (co-IP) procedures (next section). The analysis of bound forms to Lipid A by Western blot was performed first by incubation of samples with the antibody against the specific protein of interest (i.e., ApoAI). Then, a stripping procedure was performed, and the membranes were incubated with the antibody against Lipid A, which shows a band with a similar molecular weight to the protein of interest (note that free Lipid A weighs ∼10 kDa), indicative of the [Lipid A-protein] complex (which has also been demonstrated by co-IP procedures). The results of the binding between Lipid A and each apolipoprotein were expressed as the quantification of the bound form normalized by the total amount of the specific protein/apolipoprotein in this brain area.

### co-IP procedure

Co-IP is a biochemical method to precipitate a complex using target-specific antibodies. In this study, co-IP was used to study LipidA-Apo binding (protein‒protein interaction). The Lipid A antibody was first used to immunoprecipitate, and then it was immunoblotted using specific ApoAI or ApoB antibodies (Apo E was not used because no colocalization of Lipid A and ApoE was found in Western blotting). The signal obtained in the co-IP means that ApoAI or ApoB is immunoprecipitated in Lipid A, confirming the binding of Lipid A to ApoAI or ApoB.

Co-IP was performed based on a previously published protocol (37, 38). Brain samples were mechanically homogenized using 5 mm stainless steel beads in a TissueLyser LT (Qiagen®, Hilden, Germany) with 1 ml of 50 mM Tris buffer (pH = 7.4) mixed with a protease inhibitor cocktail for each brain tissue sample. The frequency used was 50 oscillations for 2 min 3 times, followed by centrifugation at 1 000 g for 10 min at 4°C. The supernatants were collected and centrifuged at 12,000 g for 30 min at 4°C. Then, the pellets were resuspended in Tris buffer at a dilution of 1:750 (w/v). Protein levels were measured and adjusted by Bradford’s method, and homogenates were mixed with Tris buffer to obtain a final concentration of 4 mg/ml.

To obtain concentrated samples from small amounts of tissue and due to the low signal intensity obtained after co-IP during the first trials, a sample pooling of two biological replicates in a group was performed. All samples were centrifuged at 12,000 g for 30 min at 4°C, and pellets were resuspended in 200 μl of RIPA buffer (R0278, Sigma‒Aldrich®, Madrid, Spain) containing protease inhibitor cocktail and incubated for 30 min with constant rotation at 4°C. Samples were centrifuged at 12,000 g for 30 min at 4°C, and supernatants were collected in Eppendorf tubes containing 10 μl of Lipid A antibody (Supplemental Table S3) and incubated overnight with constant rotation at 4°C to allow the formation of immune complexes. Twenty-five microliters of Protein A Agarose resin (P3476, Sigma‒Aldrich®, Madrid, Spain) was added to the samples and incubated with constant rotation for 2 h at 4°C to collect the immune complexes. The resin was washed three times by centrifuging at 10,000 g for 1 min at 4°C. Resins were first resuspended in 200 μl of RIPA buffer and, in the last wash, with Laemmli simple buffer containing β-mercaptoethanol, and they were analyzed by SDS‒PAGE and immunoblotted using ApoAI or ApoB antibodies (Supplemental Table S2).

### BEC determination

Ethanol levels in plasma samples were measured by the commercial Enzychrom TM Ethanol Assay Kit ECET-100 (BioAssay Systems®, Hayward, CA) according to the manufacturer’s protocol. The absorbance of each well was measured at 570 nm using a ThermoMax microplate reader (Molecular Devices®, Ramsey).

### Plasma corticosterone levels

Plasma corticosterone levels were determined by a colorimetric competitive enzyme immunoassay kit (Catalog No. ADI-900-097, Enzo Life Sciences®, Lauren, Switzerland) following the manufacturer’s instructions. Standards and plasma samples were assayed in duplicate. Absorbance was measured at 405 nm using a ThermoMax Microplate reader (Molecular Devices®, Ramsey, USA). Calculated values are expressed as nanograms of corticosterone per milliliter (ng/ml).

### Plasma LPS determination

Plasma LPS levels were determined using a commercially available kit based on ELISA following the manufacturer’s instructions (Hycult Biotech®, Uden, The Netherlands). This test is based on the ability of the endotoxin to cause intravascular coagulation in the American horseshoe crab, Limulus polyphemus. This endotoxin causes an opacity and gelation in Limulus amebocyte lysate, producing an enzymatic reaction and a yellow color. LPS was measured at 450 nm (Molecular Devices®, Ramsey). The results were obtained as endotoxin units per mL (EU/ml) and expressed as a percentage of control values.

### Determination of apolipoproteins

ApoAI, B, and E in plasma samples were measured using the sandwich-ELISA principle with a commercial assay kit (Catalog No. E-EL-R3029, E-EL-R1218, E-EL-R1230, respectively, Elabscience Biotechnology® Inc.) following the manufacturer’s instructions. The absorbance was measured at 450 nm using a ThermoMax microplate reader (Molecular Devices®, Ramsey).

### HDL and LDL levels

HDL and LDL levels were measured in rat plasma using commercially available sandwich enzyme immunoassays (SEB006Ra-96T and SEB107Ra-96T, respectively, Cloud-Clone Corp.®, TX). The final concentrations of HDL and LDL in the samples were determined by comparing the O.D. of the samples to the standard curve by measuring the color change spectrophotometrically at 450 nm wavelength.

### Statistical analyses

All data are expressed as the mean ± S.E.M. Data from ELISA kits were analyzed using a 2-way ANOVA, comparing the factors [alcohol/water] versus sex [male/female], when normality was verified; otherwise, a Kruskal-Wallis test was used. *Post hoc* comparisons (Bonferroni) were performed in case of significant interaction between factors. Homoscedasticity was checked by Barlett’s test, and data were transformed (sqrt, log_10_) when appropriate. Data from western blots of each sex were analyzed independently, comparing alcohol-treated animals versus controls by using the parametric Student’s *t* test or the nonparametric Mann‒Whitney test, due to the samples from each sex were loaded in different blots. The outliers were analyzed using Grubbs’ test. Correlations were assessed by Pearson’s and linear regression analyses. A *P* value < 0.05 was set as the threshold for statistical significance in all statistical analyses. All data were analyzed using GraphPad Prism version 8.01 (GraphPad Software, Inc., La Jolla, CA).

## Results

### Plasma LPS, LBP, and Apos (AI, B, and E) levels in male and female ethanol-intoxicated and control animals

**Fig. 1:**
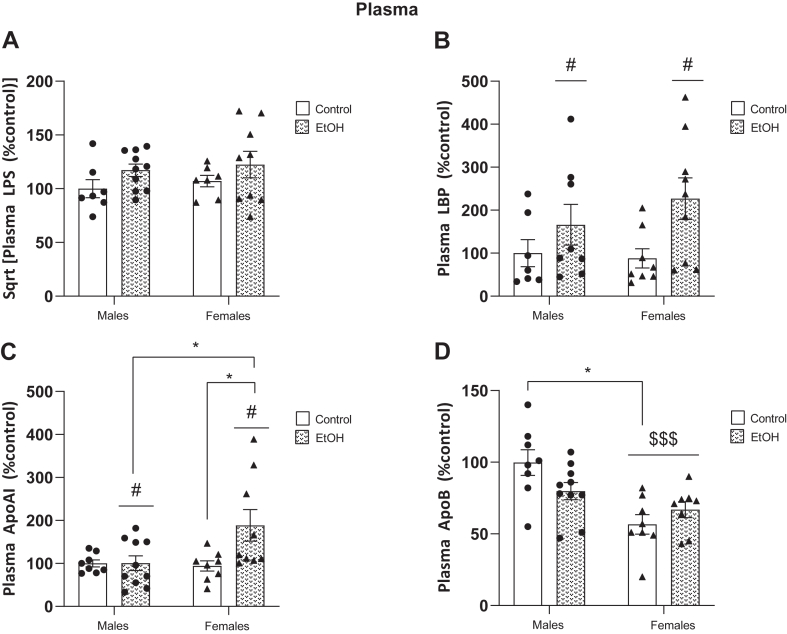
Expression of LPS, LBP, and apolipoproteins in the plasma of male and female ethanol-treated and control animals. A: Plasma LPS levels. B: Plasma LBP levels. C: Plasma ApoAI. D: Plasma ApoB. No detectable levels for plasma ApoE. All data are expressed as mean ± S.E.M. Statistical analysis: 2-way ANOVA: overall effect of ethanol: ^*#*^*P* < 0.05; overall effect of sex: ^$$$^*P* < 0.001; interaction between factors (alcohol/sex), followed by Bonferroni *post hoc* test: ∗*P* < 0.05. LBP, LPS-binding protein; LPS, lipopolysaccharide.

The levels of the different mediators were measured 3 h after the last ethanol i.g. administration and results analyzed using 2-way ANOVA in order to study differences between the four groups, including sexual differences. LPS was detectable in plasma in control animals both in male (0.491 ± 0.087 EU/ml) and female rats (0.612 ± 0.079 EU/ml). After 2-way ANOVA analyses, we did not find significant changes in plasma LPS levels (no interaction between factors: F_(1, 29)_ = 0.01027, *P* = 0.9200), but we observed an overall ethanol effect near of significance in both ethanol-treated groups (Fig. 1A; F_(1, 29)_ = 3.441, *P* = 0.0738) with no sexual differences (F_(1, 29)_ = 0.4960; *P* = 0.4869, respectively).

LBP was also detectable in plasma in all the experimental groups, and it was elevated in the ethanol-treated groups versus control groups (Fig. 1B; 2-way ANOVA, overall alcohol effect: F_(1, 28)_ = 6.436, *P* = 0.0170), disregarding of sex (F_(1, 28)_ = 0.3616; *P* = 0.5525; no interaction F_(1, 28)_ = 0.8090; *P* = 0.3761).

Regarding ApoAI levels in plasma (Fig. 1C), control males showed similar basal levels (529.15 ± 41.53 μg/ml) than control females (498.44 ± 62.68 μg/ml). 2-way ANOVA reported a significant interaction between ethanol and sex (Fig. 1C; F_(1, 31)_ = 4.376, *P* = 0.0447) with an overall ethanol effect (F_(1, 31)_ = 4.489; *P* = 0.0422) and a sex effect near of significance (F_(1, 31)_ = 3.368, *P* = 0.0761). *Post hoc* comparisons revealed that plasma ApoAI was elevated in female alcohol-treated rats versus female controls and versus alcohol-treated males (Fig. 1C, *P* < 0.05 in both cases).

Plasma ApoB (Fig. 1D) showed an overall sex effect (F_(1, 30)_ = 16.40, *P* = 0.0003) and an interaction between factors (F_(1, 30)_ = 4.762, *P* = 0.0371) after 2-way ANOVA. *Post hoc* test revealed a basal sexual dimorphism with control females showing lower levels of plasma ApoB (684.19 ± 1.18 μg/ml) than control males (1206.68 ± 107.29 μg/ml) but no specific effect of alcohol (F_(1, 30)_ = 0.4913, *P* = 0.4888, n.s.) (Fig. 1D).

ApoE levels in plasma were under detection limits in ELISA in all experimental and control groups tested.

### Plasma HDL and LDL, corticosterone, and BECs in male and female animals

Since each Apo studied in the section before is mainly incorporated into lipoproteins of different densities and according to the LPS transport or detoxification theories explained in the introduction, we quantified the plasma levels of HDL (which incorporates mainly ApoAI and ApoE) and LDL (which incorporates ApoB).

The results of plasma HDL and LDL are shown in Table 1. A 2-way ANOVA found no interaction between factors for HDL (F_(1, 31)_ = 0.6047, *P* = 0.4427) and no overall alcohol (F_(1, 31)_ = 0.7839, *P* = 0.3828) or sex (F_(1, 31)_ = 0.7870, *P* = 0.3819) effects. Regarding LDL, the 2-way ANOVA indicated an overall effect of sex (F_(1, 31)_ = 9.449, *P* = 0.0044) and no alcohol effect (F_(1, 31)_ = 2.626, *P* = 0.1152) or interaction (F_(1, 31)_ = 0.9269, *P* = 0.3431), revealing a basal sexual dimorphism with males showing elevated plasma LDL than women (Table1).

**Table 1:** Plasma HDL and LDL, corticosterone, and BELs

	Males		Females			
	Control	EtOH	Control	EtOH	Student's *t* test	2-way ANOVA
HDL (μg/ml)	517.23 ± 43.30	415.08 ± 63.69	414.98 ± 60.73	408.36 ± 68.51	---	n.s.
LDL (μg/ml)	171.10 ± 6.27	138.20 ± 15.24	119.70 ± 12.18	111.32 ± 13.02	---	Overall sex effect: ^**$$**^*P* < 0.01
Corticosterone (ng/ml)	186.53 ± 38.74	156.11 ± 15.81	165.74 ± 22.97	418.05 ± 71.83∗^**&**^	----	Overall alcohol effect: ^**#**^*P* < 0.05 Overall sex effect: ^**$**^*P* < 0.05 Interaction (*post hoc* test): ∗^&^*P* < 0.05
BEL (mg/dl)	---	101.52 ± 19.61	---	56.95 ± 17.09∗	*P* = 0.0491	---

No differences between males and females were detected in plasma HDL. An overall effect of sex was found in LDL levels, with higher levels in males. There was an interaction between alcohol and sex in the levels of corticosterone: alcohol-treated females had higher corticosterone levels than the female controls (Bonferroni *post hoc* test: ∗*P* < 0.05) and alcohol-treated males (Bonferroni *post hoc* test: ^&^*P* < 0.05). BEL (blood alcohol levels achieved 3 h last ethanol gavage) differed between male and female animals (Student's *t* test, ∗*P* < 0.05). Data are shown as the mean ± S.E.M. Statistical analysis: 2-way ANOVA: overall effect of ethanol: ^#^*P* < 0.05; overall effect of sex: ^$$^*P* < 0.001; interaction between factors (alcohol/sex), followed by Bonferroni *post hoc* test: ∗*P* < 0.05.

We additionally checked BECs and corticosterone in males and females (Table 1). The mean BECs achieved 3 h after ethanol administration in males were within the binge drinking definition, whereas females had lower BECs (<80 mg/dl; Table 1, Student's *t* test, *P* < 0.05). However, regarding corticosterone levels, an interaction between factors was found (F_(1, 30)_ = 10.01, *P* = 0.0036) with overall ethanol and sex effects (F_(1, 30)_ = 6.165; *P* = 0.0188; F_(1, 30)_ = 7.282; *P* = 0.0113, respectively). *Post hoc* comparisons revealed that corticosterone was not altered during experimental conditions in males but it was increased in female alcohol-treated animals versus female controls (*P* < 0.01). Estrous cycles in female rats were recorded during the experiment, and they are shown in the Supplemental Information (Supplemental Results 2.1, Supplemental Table S4 and Supplemental Fig. S2).

### Detection of LPS components (Lipid A and Core) in the brains of male and female alcohol-intoxicated and control animals

**Fig. 2:**
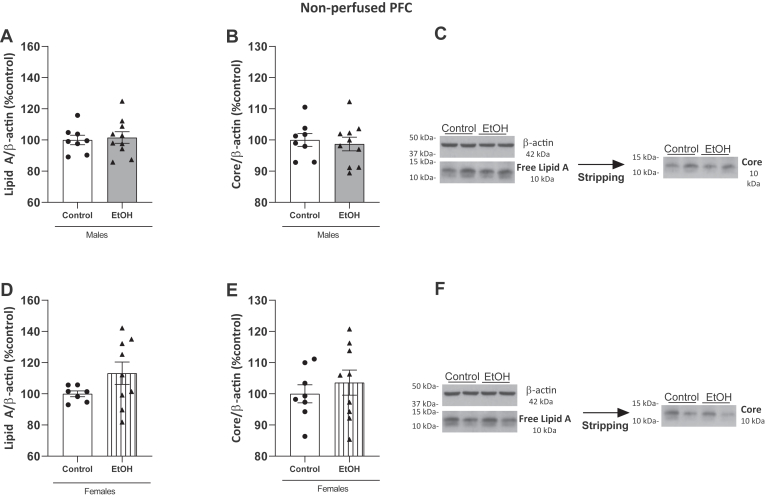
Detection of the LPS components Lipid A and core in prefrontal cortex by Western blotting. The *upper* panel shows data in ethanol-treated (n = 10) and control (n = 8) males and the lower panel data in ethanol-treated (n = 9) and control (n = 8) females. A: Expression of Lipid A in males in PFC. B: Expression of the core element of LPS in males. C: Representative immunoblots of Lipid A and core from the same gel in males. D: Expression of Lipid A in females in PFC. E: Expression of core element in females. F: Representative immunoblots of Lipid A and core from the same gel in females. Western blot data were normalized by β-actin and expressed as a percentage of change over controls. Results represent the mean ± S.E.M. of two technical replicates. No differences were observed between groups. Student’s *t* test. LPS, lipopolysaccharide.

The components of LPS, Lipid A and Core, were measured in nonperfused PFC (Fig. 2) and cerebellum (Supplemental Fig. S3) of male and female animals treated with alcohol binges and their control groups. Interestingly, both Lipid A (free form, see results Screening study of the binding of Lipid A to different molecules in the PFC: Pilot study with TLR4) and Core were detectable and measurable within the brain (including cerebral blood flow) of males and females in both experimental groups and in both brain structures. Samples in western blots were uploaded independently by sex and, thus, results are graphed as percentage of change over each respective control group and analyzed accordingly.

Figure 2 shows the expression of LPS components in the PFC of male (upper panel) and female (lower panel) animals. There were no significant differences in the expression of Lipid A or Core levels (Fig. 2A, B; t_(16)_ = 0.3174, *P* = 0.7551; t_(16)_ = 0.4200, *P* = 0.6801, respectively) between the ethanol and control groups in males. Representative blots are shown in Fig. 2C.

In females, there were no changes in Lipid A and Core between the control and alcohol-treated groups (Fig. 2D, E; Mann‒Whitney U = 21; *P* > 0.05, n.s. ; t_(15)_ = 0.7107, *P* = 0.4881, respectively). Representative blots are shown in Fig. 2F.

The results in the cerebellum were not significant between groups and are shown in the Supplementary Results (Supplemental Fig. S3).

### Screening study of the binding of Lipid A to different molecules in the PFC: Pilot study with TLR4

After studying the expression of Lipid A and Core in the PFC in their free forms, our goal was to investigate the possible binding of these elements to different apolipoproteins within the brain. We chose Lipid A (the LPS domain considered endotoxin) for this colocalization study.

**Fig. 3:**
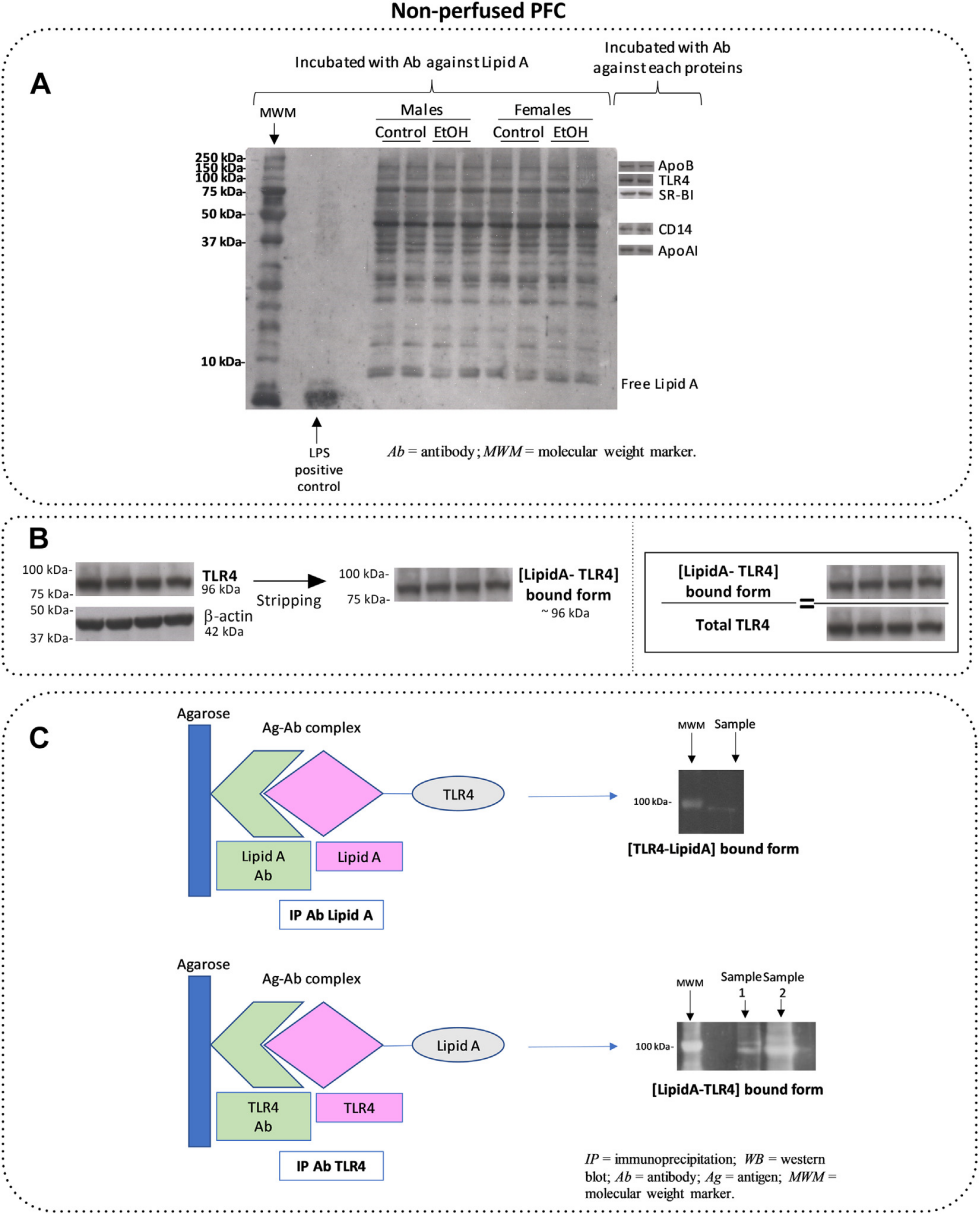
Screening study of Lipid A bound to different molecules in PFC: pilot study with TLR4. A: Representative immunoblot of a membrane from a 18% gel incubated with antibody against Lipid A, where Lipid A showed expression at different molecular weights (left side). On the right side, samples of immunoblots which correspond with receptors and apolipoproteins which Lipid A may bind to. B: Representation of the binding of Lipid A to TLR4. The ratio [LipidA-TLR4]-bound form is an indirect measurement of colocalization of proteins. [LipidA-TLR4]-bound form was detected at 96 kDa and normalized by total TLR4. Blot images in the far-right-side are reused from the left and middle-side in order to represent the normalization (ratio) process. C: Left panel in (C) is a schematic representation of the co-IP process as a direct measure of colocalization of Lipid A and TLR4. Upper panel represents the immunoreactivity obtained from Lipid A immunoprecipitated incubated with an antibody against TLR4. Down panel shows the immunoreactivity obtained from TLR4 immunoprecipitated incubated with an antibody against Lipid A. co-IP, coimmunoprecipitation.

In a pilot study, we detected that Lipid A showed expression by Western blot at different molecular weights, which corresponded with receptors and apolipoproteins to which it may bind, as suggested in previous publications (31). Thus, Fig. 3A shows the complete profile of Lipid A expression by Western blot when incubated with the antibody against Lipid A. Figure 3A shows a Lipid A positive control (15 μg of *E. coli* LPS (O111:B5)), and the rest of the bands are samples of male control and ethanol-treated animals. The free Lipid A (not bound form) was visualized in a band at approximately 10 kDa. However, the antibody against Lipid A also showed other immunoreactivities at different molecular weights, indicative of the well-known binding of LPS to other molecules, as suggested before (31). Specifically, we detected clear bands at ∼31 kDa, ∼48 kDa, ∼75 kDa, ∼96 kDa, and ∼210 kDa, which may correspond to the binding of Lipid A to ApoAI, CD14, SR-B1, TLR4, and ApoB, respectively. Immunoblots of each of those proteins incubated with each specific antibody in each case are shown at the right of the panel in Fig. 3A.

To determine whether Lipid A was bound to some of these components, we used two approaches: 1) we checked the expression of each mentioned protein at the specific molecular weight by incubating first with the antibody against Lipid A and then with the antibody of interest, and vice versa; 2) we confirmed by co-IP the binding of the two proteins of interest.

Thus, as an example, Fig. 3B, C represent the binding of Lipid A to TLR4. Figure 3B shows the expression of TLR4 (band at ∼96 kDa). The membrane was stripped and then incubated with the antibody against Lipid A, which also detected a band near the same molecular weight. The results of the [LipidA-TLR4]-bound form were normalized to the total expression of TLR4 (free + bound form; incubation with antibody against TLR4) in the PFC (Fig. 3B).

The binding of Lipid A to TLR4 was confirmed twice by co-IP. First, we precipitated the conjugate using an antibody against Lipid A, and then the complex was incubated with the antibody of interest, in this specific case, TLR4 (Fig. 3C, upper panel). To double check this binding, another set of samples was prepared, and the conjugate was precipitated using an antibody against TLR4 first. Then, the complex was visualized by analysis of the immunoreactivity against Lipid A (Fig. 3C, lower panel).

In this pilot study, we showed that our approach of binding of Lipid A and TLR4 by Western blotting was confirmed by co-IP. In a new study, we used both Western blotting and co-IP to check the binding of Lipid A to TLR4 and different apolipoproteins and their receptors, and the results are presented in the next sections. Co-IP was used as qualitative confirmation of each specific binding of proteins (conjugate first precipitated by Lipid A antibody and then the complex incubated with the antibody of the protein of interest), and we used Western blot analyses to quantify the samples in our study, since the co-IP is limiting tissue technique that needs pooled samples (see Materials and methods section).

In the next sections, we report the studies of the binding of Lipid A with TLR4, ApoAI, ApoB, and ApoE in the different experimental groups.

### TLR4 expression and quantification of LipidA-TLR4-bound form in male and female rats under alcohol or control conditions

**Fig. 4:**
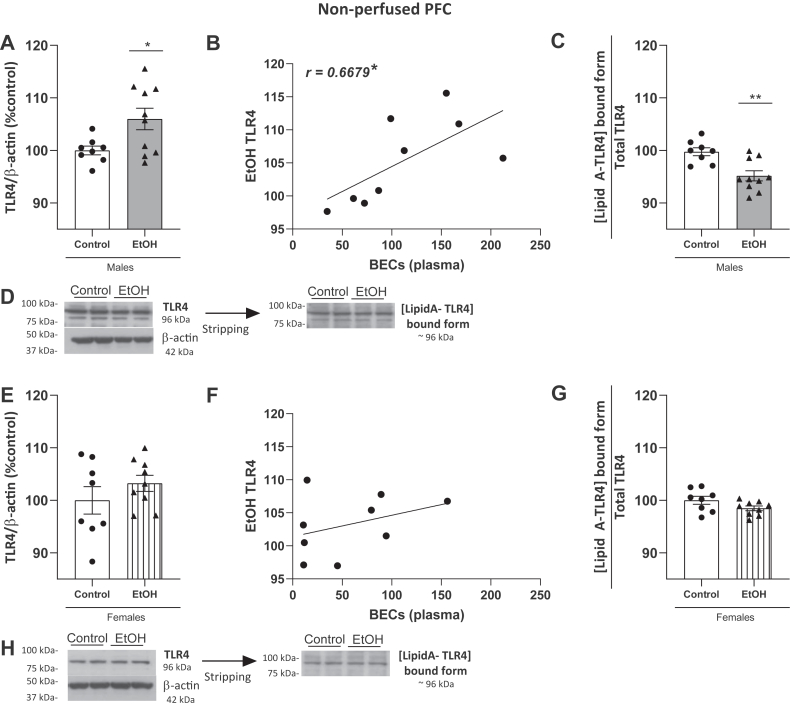
TLR4 and [LipidA-TLR4]-bound form expression by Western blotting and TLR4-BECs linear regression in PFC. The upper panel shows data in ethanol-treated versus control males and the lower panel data in ethanol-treated versus control females. A: TLR4 levels were increased in ethanol-treated males. B: Linear regression between TLR4 in PFC and BECs in plasma in males. The trend line shows the regression analyses for the ethanol-treated group. BECs in plasma were positively correlated with the TLR4 levels in PFC. C: The ratio [LipidA-TLR4]-bound form in males, as indirect measurement of colocalization of proteins, were decreased in ethanol-treated group. Lipid A was detected at 96 kDa and normalized by total TLR4. D: Representative immunoblots of total TLR4 and [LipidA-TLR4]-bound form from the same gel of males. E: Expression of total TLR4 in females. F: Linear regression between TLR4 in PFC and BECs in plasma females. G: The ratio [LipidA-TLR4]-bound form in females. H: Representative immunoblots of total TLR4 and [LipidA-TLR4]-bound form from the same gel in females. Western blot data were normalized by β-actin and expressed as a percentage of change over controls. Results represent the mean ± S.E.M. of two technical replicates. Differences from control group: ∗*P* < 0.05, ∗∗*P* < 0.01 (Student’s *t* test or Pearson’s coefficient correlation *r*).

We used both Western blotting and co-IP to check the binding of Lipid A to TLR4 and different apolipoproteins. Figure 4A represents the total expression of TLR4 in the PFC of male rats under ethanol or control conditions. We observed that TLR4 was upregulated in ethanol-treated male rats (Fig. 4A; t_(16)_ = 2.484, *P* = 0.0244), whereas there was no significant effect in females. Notably, at the time-point of tissue extraction (3 h after the last alcohol binge), males showed higher BECs than females (Table 1), and we found a positive correlation between BECs and TLR4 expression in the PFC in male animals (Fig. 4B, *r* = 0.6679, *P* < 0.05) that was not found in females. The study of binding between TLR4 and Lipid A is represented in Fig. 4C for males. Data are expressed as [LipidA-TLR4]-bound form normalized by the expression of total TLR4. The results indicate that the [LipidA-TLR4]-bound form is decreased in male ethanol-treated animals (Fig. 4C; t_(16)_ = 3.589, *P* = 0.0025). Figure 4D shows the representative blots for Western blot analyses in males.

The bottom panel of Fig. 4 shows the same parameters in female animals. As commented before, no significant effect was found in TLR4 expression in female ethanol rats compared to controls in the PFC (Fig. 4E; t_(15)_ = 1.098, *P* = 0.2897, n.s.), no correlation of TLR4 with BECs (Fig. 4F, *r* = 0.3589, *P* > 0.05), and no binding of TLR4-Lipid A was found in females (Fig. 4G; t_(15)_ = 1.769, *P* = 0.0971, n.s.). Figure 4H shows representative blots in female animals.

### Expression of ApoAI, [LipidA-ApoAI] aggregates, and SR-BI receptors in the PFC in male and female ethanol-treated and control animals

**Fig. 5:**
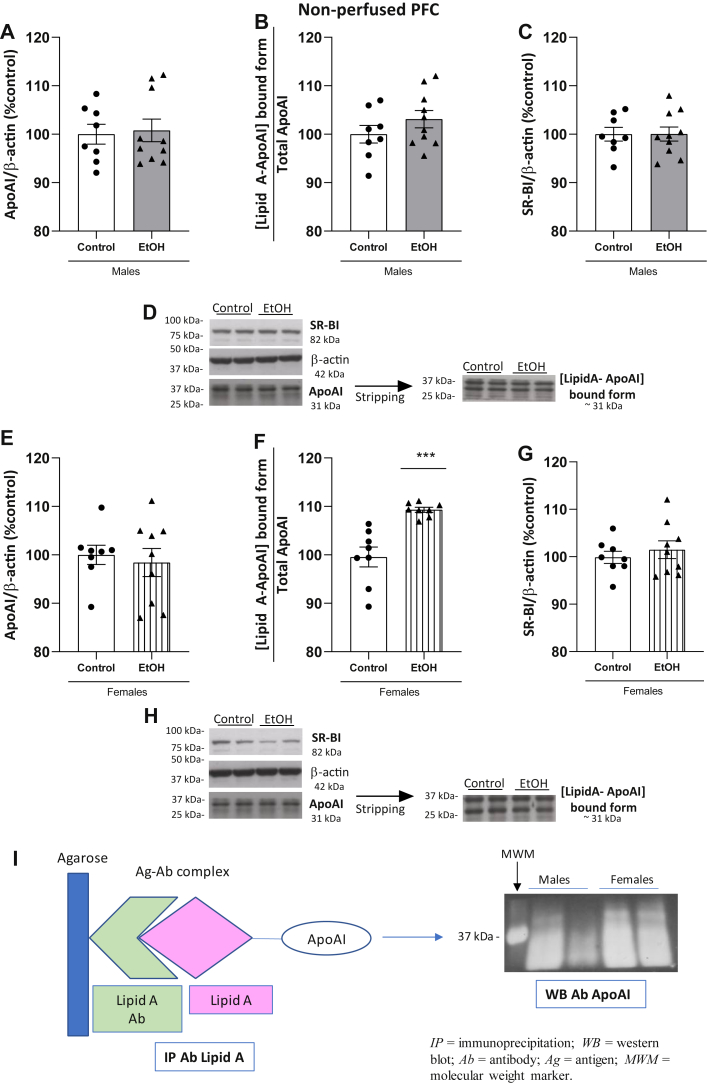
Expression of ApoAI and [LipidA-ApoAI] by Western blotting and its receptor SR-BI in PFC. The upper panel shows data in ethanol-treated (n = 10) and control (n = 8) males and the lower panel data in ethanol-treated (n = 9) and control (n = 8) females. A: Expression of total ApoAI in males. B: The ratio [LipidA-ApoAI]-bound form in males, as indirect measurement of colocalization of proteins (Lipid A detected at 31 kDa and normalized by total ApoAI). C: Expression of SR-BI in males. D: Representative immunoblots of total ApoAI, [LipidA-ApoAI]-bound form, and SR-BI in males from the same gel. E: Expression of total ApoAI levels in females. F: The ratio [LipidA-ApoAI]-bound form in female, as indirect measurement of colocalization of proteins, was increased in ethanol-treated females. Lipid A was detected at 31 kDa and normalized by total ApoAI. G: Expression of SR-BI levels in female rats. H: Representative immunoblots of total ApoAI, [LipidA-ApoAI]-bound form, and SR-BI in females from the same gel. I: Colocalization of Lipid A and ApoAI by co-immunoprecipitation (co-IP). Left panel in (I) is a schematic representation of the co-IP process. Co-IP is a direct measure of colocalization of Lipid A and ApoAI. Right panel shows a representative image of the co-IP. Results were obtained by pool of samples in the same experimental group and are descriptive. Quantification of data was done by Western blotting, and it is shown in (B) and (F). Results represent the mean ± S.E.M. of two technical replicates. Differences from control group: ∗∗∗*P* < 0.001 (Student’s *t* test or Mann-Whitney (3F)). SR-BI, scavenger receptor class B type 1.

ApoAI was detectable in the PFC of control and ethanol-treated animals by Western blotting (Fig. 5). In male rats, there were no differences between the alcohol and control groups in total PFC ApoAI levels (Fig. 5A; t_(16)_ = 0.2420, *P* = 0.8119). Regarding the [LipidA-ApoAI]-bound form, we did not observe significant differences between groups (Fig. 5B; t_(16)_ = 1.209, *P* = 0.2442). The data showed that SR-BI was detectable in this structure by Western blot, with no changes between the ethanol and control groups (Fig. 5C; t_(16)_ = 0.02170, *P* = 0.9830). Figure 5D shows the representative blots for these proteins in males.

In female rats, whereas no differences in total ApoAI levels were detected in the PFC (Fig. 5E, t_(15)_ = 0.4364, *P* = 0.6687), we observed an increase in the [Lipid A-ApoAI]-bound form in the ethanol group compared to controls (Fig. 5F; Mann‒Whitney, U = 0, *P* < 0.001). Expression of the ApoAI receptor SR-BI was not altered in females (Fig. 5G; t_(15)_ = 0.6976, *P* = 0.4961). Blots are represented in Fig. 5H for female animals.

Figure 5I shows a representative diagram of the precipitated complex in the co-IP procedure using pooled samples (left panel) and a representative image of the confirmation of the binding between Lipid A and ApoAI by co-IP in the PFC (right panel).

### Expression of ApoB, [Lipid A-ApoB] aggregates, and LDLr in the PFC of male and female ethanol-treated and control animals

**Fig. 6:**
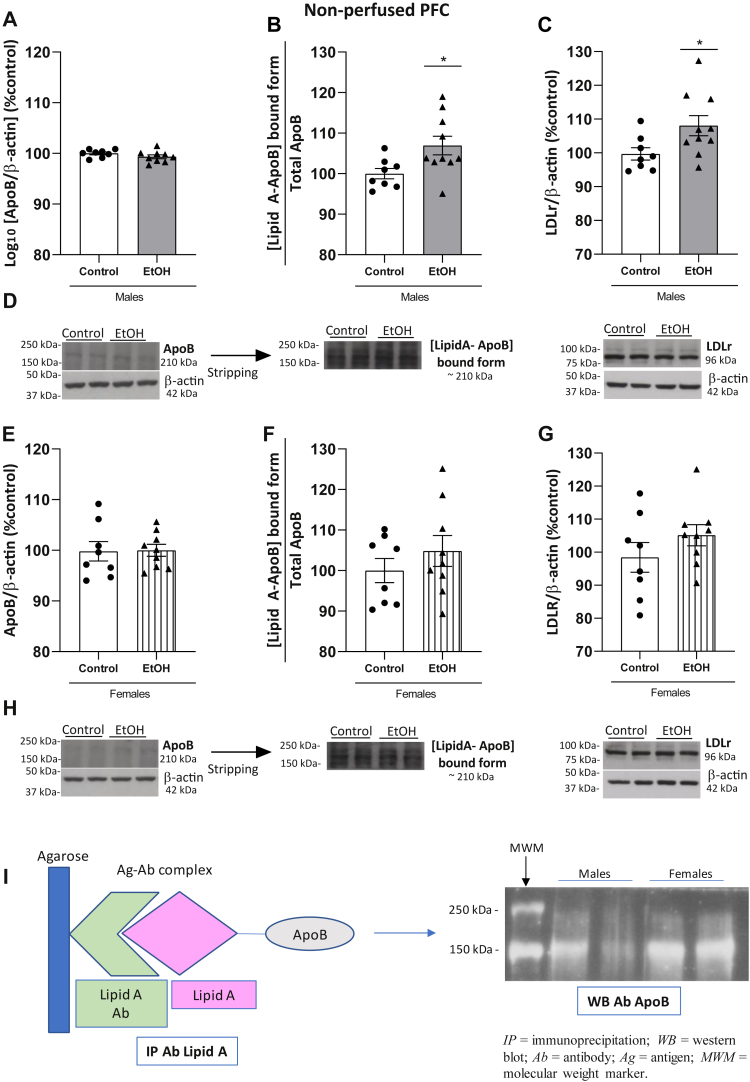
Expression of ApoB and [LipidA-ApoB] by Western blotting and its receptor LDLr in PFC. The upper panel shows data in ethanol-treated (n = 10) and control (n = 8) males and the lower panel data in ethanol-treated (n = 9) and control (n = 8) females. A: Expression of total ApoB in males. B: The ratio [LipidA-ApoB]-bound form in males, as indirect measurement of colocalization of proteins, was increased in ethanol-treated group. Lipid A was detected at 210 kDa and normalized by total ApoB. C: LDLr levels were increased in ethanol group in males. D: Representative immunoblots of total ApoB, [LipidA-ApoB]-bound form, and LDLr in males from different gels. E: Expression of total ApoB levels in females. F: The ratio [LipidA-ApoB]-bound form in female, as indirect measurement of colocalization of proteins (Lipid A was detected at 210 kDa and normalized by total ApoB). G: Expression of LDLr levels in female rats. H: Representative immunoblots of total ApoB, [LipidA-ApoB]-bound form, and LDLr in females from different gels. I: Colocalization of Lipid A and ApoB by co-IP. Left panel in (I) is a schematic representation of the co-IP process. Co-IP is a direct measure of colocalization of Lipid A and ApoB. Right panel shows a representative image of the co-IP. Results were obtained by pool of samples in the same experimental group and are descriptive. Quantification of data was done by Western blotting, and it is shown in (B) and (F). Results represent the mean ± S.E.M. of two technical replicates. Differences from control group: ∗*P* < 0.05 (Student’s *t* test). co-IP, coimmunoprecipitation; LDL, low-density lipoprotein; LDLr, LDL receptor.

ApoB levels were detectable in the PFC in all experimental groups, and quantifiable analyses are shown in Fig. 6. There were no significant differences in total ApoB levels between the alcohol and control groups in males (Fig. 6A; t_(15)_ = 1.381, *P* = 0.1876). However, we observed a clear increase in the [LipidA-ApoB]-bound form in ethanol-treated male animals versus controls (Fig. 6B; t_(16)_ = 2.448, *P* = 0.0263), and interestingly, levels of the ApoB receptor LDLr in the PFC were also upregulated in the ethanol group in males (Fig. 6C; t_(16)_ = 2.253, *P* = 0.0387). Figure 6D shows representative blots for these proteins in male rats.

In females, there were no significant changes between groups in total ApoB levels (Fig. 6E; t_(15)_ = 0.09525, *P* = 0.9254), the [LipidA-ApoB]-bound form (Fig. 6F; t_(15)_ = 0.9816, *P* = 0.3419), or the expression of LDLr (Fig. 6G; t_(15)_ = 1.235, *P* = 0.2360). Figure 6H shows the representative blots for these proteins in females.

The binding of Lipid A with ApoB was confirmed by visualization of the immunoprecipitated complex by co-IP in pooled samples (Fig. 6I).

### Expression of ApoE, [Lipid A-ApoE] aggregates, and ApoER2 in the PFC of male and female ethanol-treated and control animals

**Fig. 7:**
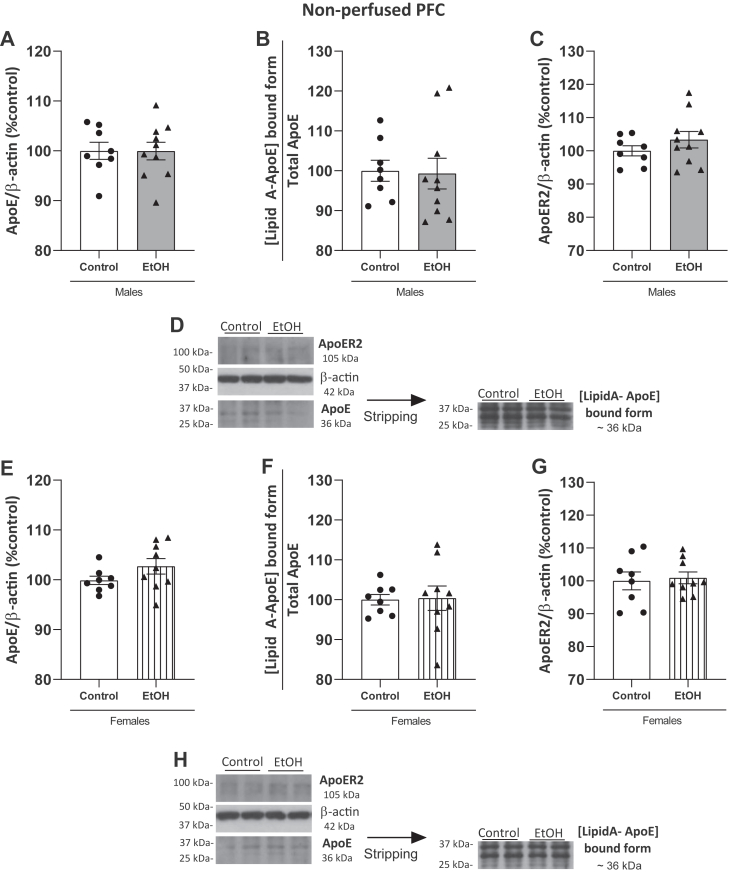
Expression of ApoE and [LipidA-ApoE] by Western blotting and its receptor ApoER2 in PFC. The upper panel shows data in ethanol-treated (n = 10) and control (n = 8) males and the lower panel data in ethanol-treated (n = 9) and control (n = 8) females. A: Expression of total ApoE in males. B: The ratio [LipidA-ApoE]-bound form in males, as indirect measurement of colocalization of proteins (Lipid A was detected at 36 kDa and normalized by total ApoE). C: ApoER2 levels in males. D: Representative immunoblots from the same gel of total ApoE, [LipidA-ApoE]-bound form, and ApoER2 in males. E: Expression of total ApoE levels in females. F: The ratio [LipidA-ApoE]-bound form in female, as indirect measurement of colocalization of proteins (Lipid A was detected at 36 kDa and normalized by total ApoE). G: Expression of ApoER2 levels in female rats. H: Representative immunoblots from the same gel of total ApoE, [LipidA-ApoE]-bound form, and ApoER2 in females. Western blot data were normalized by β-actin and expressed as a percentage of change over controls. Results represent the mean ± S.E.M. of two technical replicates. There were no differences between groups (Student's *t* test, *P* > 0.05). ApoER2, ApoE receptor 2.

Although ApoE levels in plasma were detected by ELISA under our experimental conditions (see Results Plasma LPS, LBP, and Apos (AI, B, and E) levels in male and female ethanol-intoxicated and control animals), they were clearly detected in the PFC in both the control and ethanol groups (Fig. 7), as expected due to its astrocyte origin.

Figure 7 shows that there were no differences between the alcohol and control groups in total ApoE levels, in the [LipidA-ApoE]-bound form (Fig. 7A, B; t_(16)_ = 0.01856, *P* = 0.29854; t_(16)_ = 0.1418, *P* = 0.8890, respectively) or in ApoER2 expression (Fig. 7C; t_(16)_ = 1.082, *P* = 0.2953) in the PFC of males. Blots are represented in Fig. 7D.

Similarly, no differences were found in the total form of ApoE, the [LipidA-ApoE]-bound form, and ApoER2 expression in females (Fig. 7E–G; t_(15)_ = 1.553, *P* = 0.1413; Mann‒Whitney U = 34; *P* > 0.05; t_(15)_ = 0.2900, *P* = 0.7758 n.s., respectively). Representative blots in Fig. 7H.

### Detection of LPS components (Lipid A and core), TLR4, and Apos in the cerebellum of male and female ethanol-treated and control animals

The whole study was repeated in the cerebellum (nonperfused animals), both in the vermis and in the cerebellar hemispheres (Hcb) (Supplemental Results 2.2). As mentioned before, Lipid A, Core, and TLR4 were also detectable within the cerebellum of male and female animals, with no differences between experimental groups (Supplemental Results 2.2, Supplemental Fig. S3).

The expression of ApoAI and ApoB was also checked in the cerebellum, together with their receptors and their bound forms to Lipid A (Supplemental Results 2.2). No significant differences were found between experimental groups (Supplemental Figs. S4 and S5 for studies with ApoAI and ApoB, respectively).

The study with ApoE is represented in Supplemental Fig. S6 (Supplemental Results 2.2) and showed sex differences in the expression of ApoER2, with upregulation in ethanol-treated males versus controls (Supplemental Fig. S6C; Mann‒Whitney U = 10, *P* < 0.05) and downregulation in ethanol-treated females (Supplemental Fig. S6G; t_(14)_ = 3.133, *P* = 0.0073).

### Analyzing the contribution of cerebral blood flow to the presence of bacterial products within the brain

This study investigated the presence of bacterial components in their free form or bound to apolipoproteins within brain areas affected by alcohol, such as the PFC and cerebellum. Although it was not a fundamental objective of the present study, we performed a pilot experiment to ascertain the influence of blood flow within the brain in the changes observed. In this pilot study, we compared perfused (blood removed) and nonperfused female control rats regarding the presence of free Lipid A or its aggregates with ApoAI or TLR4.

**Fig. 8:**
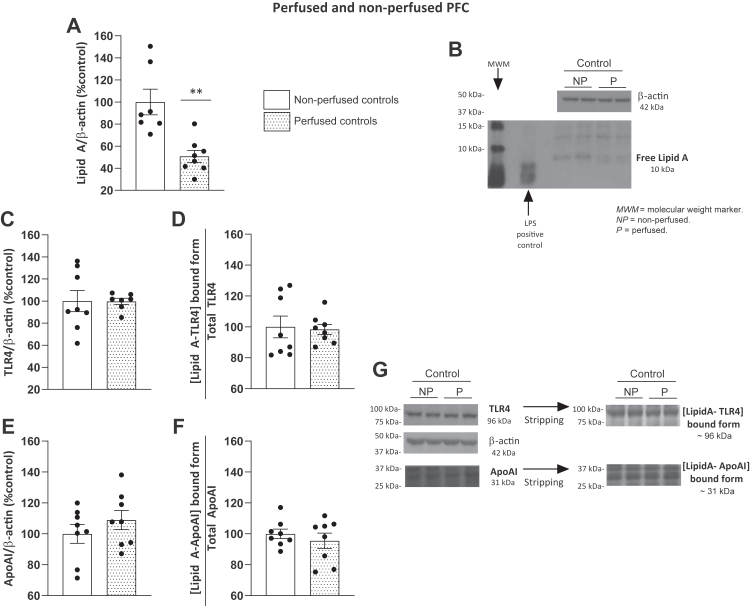
Presence of free Lipid A and [LipidA-protein] aggregates in the PFC of perfused and nonperfused female controls. A: Expression of free Lipid A. B: Representative immunoblots of Lipid A. C: TLR4 levels. D: Ratio [LipidA-TLR4]-bound form. Lipid A was detected at 96 kDa and normalized by total TLR4. E: ApoAI levels. F: Ratio [LipidA-ApoAI]-bound form Lipid A was detected at 31 kDa and normalized by total ApoAI. G: Representative immunoblots of total TLR4, total ApoAI, [LipidA-TLR4], and [LipidA-ApoAI]-bound forms from the same gel. Western blot data were normalized by β-actin and expressed as a percentage of change over nonperfused controls. Results represent the mean ± S.E.M. of two technical replicates. Differences between groups: ∗∗*P* < 0.01 (Student’s *t* test).

Figure 8A shows that free Lipid A levels were lower when the blood was removed from the animals compared with nonperfused rats (t_(13)_ = 4.020; *P* = 0.0015), according to the well-known peripheral origin of LPS. However, levels of free Lipid A were still detectable in perfused animals (Fig. 8A). Interestingly, we did not observe differences in the LipidA-TLR4 aggregates (Fig. 8C, D; Mann‒Whitney, U = 24, *P* > 0.05; t_(14)_ = 1.022; *P* = 0.3239) or LipidA-ApoAI (Fig. 8E,F; t_(14)_ = 0.2082; *P* = 0.8381; t_(14)_ = 0.7757; *P* = 0.4508) in the PFC of perfused versus nonperfused animals. These results suggest that an important percentage of Lipid A is retained in the blood vessels within the PFC but the aggregated forms with receptors or apolipoproteins are present within the cerebral blood vessel structures or even in the brain parenchyma, as suggested before by immunohistochemistry procedures (31).

## Discussion

This groundbreaking study reports the presence of parts of LPS bound to apolipoproteins in the brain of animals exposed to alcohol binge intoxications, being the apolipoprotein involved in the aggregate different in male and female rats. Specifically, we detected the presence of Lipid A, the endotoxic component of LPS, bound to ApoAI in the PFC of female rats exposed to alcohol, whereas Lipid A was found bound to ApoB in male ethanol-treated animals, which also showed increases in LDLr (ApoB/LDL main receptor) in the PFC. ApoAI was also clearly upregulated in the plasma of female ethanol-treated animals versus controls, an effect that was not observed in males. Interestingly, males in the ethanol group showed an upregulation of TLR4, a signature of neuroinflammation, in the PFC versus controls, which was not observed in females, and a reduced binding of TLR4 to Lipid A. These alterations were not observed within the cerebellum. These results suggest the existence of a differential apolipoprotein-mediated mechanism to bind LPS components in the brain of male and female animals which underwent alcohol binge intoxications. Implications for alcohol-induced neuroinflammation in the PFC are discussed below.

It is well known that alcohol intoxications induce an increase in plasma LPS due to leaky gut and liver clearance downregulation (7, 11, 15, 39, 40, 41). In the present study, despite a tendency in an overall alcohol effect, the LPS increase did not reach statistical significance, which was surprising since we previously reported LPS increases under the same binge procedure (10, 11, 15), and it is probably attributed to the slightly younger age of these animals compared with the mentioned studies. We observed, nonetheless, clear significant elevations in LBP both in females and males in response to ethanol binges, which has also been considered a marker of bacterial translocation and inflammation (42). In any case, several components of the LPS molecule, such as Lipid A and Core, were detected in the PFC and cerebellum of both sexes in the current study, with no significant differences between the control and ethanol groups. We performed a pilot study demonstrating that in perfused control animals (blood removed), the free Lipid A was lower than in intact (nonperfused) brains. These results suggest that a percentage of the Lipid A observed in this study is located outside of the BBB and other percentage may infiltrate it. This result is in agreement with mechanistic studies perfusing I-LPS where it was found that half of the I-LPS associated with brain permeated the BBB entering the parenchyma space and about half was sequestered by the capillary bed into endothelial cells (30). The Lipid A could be located at the luminal (blood-facing) or abluminal (brain-facing) side of the brain endothelial cells, but this information was not an objective of the present study. It is known that LPS may bind receptors located in brain endothelial cells, including TLR4, inducing the release of proinflammatory cytokines, and this release can occur at either side of the brain-endothelial cells (43). However, we observed that the aggregates were maintained in perfused animals at similar levels than in nonperfused rats, suggesting that LipidA-apolipoprotein bound forms could infiltrate the brain, as we reported in a previous immunohistochemical study (31). Interestingly, in the ethanol groups, Lipid A was bound to different apolipoproteins to a greater extent than in control animals and the apolipoprotein to which Lipid A binds differs in male and female animals. These results may open new avenues to the understanding of the crosstalk among alcohol, LPS, and neuroinflammation.

ApoAI is mainly synthesized in the liver and intestine (44) and works to remove excess cholesterol from cells and send LPS to the liver for its elimination (45, 46). In our study, we did not observe basal differences in the amount of ApoAI in plasma between male and female control animals, although it has been reported that women appear to have higher plasma HDL-ApoAI levels under physiological conditions than men (47). However, we found an increase in plasma ApoAI levels in the female ethanol group versus female controls, despite no changes in plasma HDL were found. This is in agreement with recent studies indicating that, rather than changes in HDL plasma levels, what varies the HDL functionality under pathological conditions is the HDL-protein cargo (i.e., ApoAI) (48). Plasma LDL, which transports mainly ApoB, plays an important role in lipid transport (45, 49), and it appears to be higher in men than in women (47). In our study, male animals also had higher plasma LDL levels than females, disregarding of the experimental treatment. In humans, moderate alcohol consumption has been associated with increases in plasma HDL levels (50) in a dose-dependent fashion and turnover of ApoAI (51), although recent studies suggest that long-term alcohol consumption may decrease HDL serum levels in women (52). Reports about the effect of binge drinking on plasma lipoproteins are very scarce in the field, with some reporting increases in plasma HDL and decreases in LDL profiles in heavy drinkers (53). Our results highlight one of the sex differences found in this study, since ApoAI plasma levels were not increased in males under ethanol intoxications. The higher levels of plasma ApoAI found in female animals of the ethanol group could indicate that females activate an ApoAI-mediated protective mechanism to neutralize LPS, since it is known that HDL, which integrates mainly ApoAI in the periphery, binds LPS and helps in its transport to the liver for elimination (46, 54).

ApoAI is also naturally present in the brain since it is known that it crosses the BBB back and forth from the circulation to the brain (55, 56), and it has been found in the CSF (57, 58, 59). In this study, we detected ApoAI in the PFC and cerebellum of both male and female rats and observed that Lipid A was specifically bound to ApoAI in the PFC of females exposed to alcohol versus controls. This effect was not observed in male animals. The upregulation of the LipidA–ApoAI complex in the PFC is parallel with an absence of TLR4 overexpression in females exposed to ethanol, which could be interpreted in different ways, as will be discussed below. As mentioned before, the wash out of the vascular space did not change the expression of LipidA-apolipoprotein aggregates within the brain in our pilot study, suggesting that they may infiltrate the brain. Similar aggregates were previously found in the blood-brain interfaces (ie tanycyte-like cells, ependymal cells, and brain-endothelial cells) but also within the cerebral parenchyma, such in astrocytes in the medulla oblongata (31). It is very possible that the Lipid A-apolipoprotein aggregates are part of the BBB (ie endothelial cells) and have a role in the vascular homeostasis, affecting proinflammatory signals at one or the other side of the barrier (28, 43). Thus, there are several possibilities to explain the Lipid A-ApoAI binding in the PFC: 1) the elevated levels of peripheral ApoAI found in the females of the ethanol group would bind Lipid A in the plasma, and the complex is transported to the blood-brain interfaces or brain parenchyma within the PFC; 2) the aggregate (Lipid A and ApoAI joined form) takes place within the BBB structures (ie endothelial cells) in the PFC at the luminal (blood-facing) or abluminal (brain-facing) side of it. Alternatively, both possibilities could be happening at the same time, and, in any case, these results would suggest that LPS components may signal in the PFC, but not in the cerebellum, after alcohol binge intoxications helped by a mechanism dependent on apolipoproteins. These hypotheses need further confirmation in future studies, as well as the precise mechanisms and the functional consequences of the binding. In any case, SR-BI appears to be responsible for HDL internalization and transcytosis across the BBB (60), but its participation in the processes described here is uncertain, since no changes in the expression of SR-BI were observed in any condition in this study.

The sequestration of Lipid A by ApoAI in the female brain under ethanol intoxications could be interpreted as a mechanism to protect it against neuroinflammation, preventing a LipidA-mediated activation of TLR4 signaling. In this line and as mentioned before, LipidA-ApoAI binding in the PFC was increased in alcohol-administered female rats, where no TLR4 signature (sign of neuroinflammation) was found. However, in males, alcohol intoxications induced a clear upregulation of TLR4 in the PFC, which was also observed in previous studies (13, 15, 61), and no binding of Lipid A with ApoAI was found. The anti-inflammatory actions of ApoAI have been described in several conditions, such as sepsis (62), and decreases in plasma ApoAI have been associated with the severity of Alzheimer's disease (48). Clearly, further research is needed to ascertain a possible protective role of ApoAI in the female brain under alcohol conditions. Intriguingly, in a very recent study, Radford-Smith and colleagues showed that intraperitoneal administration of HDL together with LPS in mice shuttles the endotoxin LPS to the brain and promotes neuroinflammation, whereas the co-administration of LDL with LPS has anti-neuroinflammatory properties (63). The binding of Lipid A with ApoAI could then be interpreted as a protective strategy to capture LPS, in line with most of the studies, or, counterintuitively, as a strategy to help LPS access the brain, as suggested elsewhere (63). Indeed, it has been suggested that lipoproteins may act with a double function: they may capture and clear LPS from blood and tissues, but they may induce inflammatory responses elsewhere (63). The dance between LPS and the lipoprotein subclasses is very complex since, although LPS displays greater affinity for HDL, it can be transferred from HDL (ApoAI) to LDL (ApoB) in response to an acute-phase response to infection (27). This is in accordance with our study, since we observed that in alcohol-treated animals, Lipid A is bound to ApoAI in the PFC of females, whereas it is bound to ApoB in males, who showed a clearer neuroinflammatory response in the PFC. Interestingly, one of the molecules in charge of this transfer of LPS from HDL to LDL is plasma LBP (27), which was found to be increased in all ethanol-treated animals versus controls. LBP is considered an even stronger neutralizing molecule against LPS-induced inflammation than ApoAI (64), suggesting that the female ethanol-treated rats in this study were double protected by elevations in both ApoAI and LBP plasma levels.

Unlike ApoAI, it is believed that ApoB cannot cross the BBB (56), and it is mainly synthesized in the liver. However, some studies have confirmed the presence of ApoB in brain endothelial cells in mice (65), suggesting that ApoB could cross the BBB indeed. Other studies have shown that brain ApoB bound to αβ plaques in transgenic Alzheimer’s disease mice (66, 67). Interestingly, LDLr is expressed in neurons and glial cells (45) and may facilitate ApoB passage from blood to brain by transcytosis (68). Here, we detected an upregulation of LDLr expression in the PFC of males that underwent alcohol administrations, and these animals also showed an increase in the LipidA-ApoB-bound form compared to controls. Altogether, these data indicates that the Lipid A bound a specific apolipoprotein in males and females under alcohol intoxications, and this result deserves further investigation. It is possible that differences in the magnitude of the acute-phase response induced by alcohol in males and females in our study account for this effect, since LPS may be exchanged between lipoprotein subclasses according to the neuroimmune capacity, as discussed above.

We also checked the presence of ApoE, one of the major apolipoproteins in the CNS, which is highly expressed by astrocytes but also by oligodendrocytes and microglia (69). ApoE is involved in the transport of cholesterol and other lipids through the bloodstream and the CNS (57, 59), and the ApoE4 isoform has been widely studied for its participation in neuroinflammation and cognitive decline (70) in several neurological disorders (71, 72), including alcohol abuse (73, 74). In our study, there were no differences in the expression of ApoE between experimental groups or its binding to Lipid A in the PFC, both in males and females. We did not distinguish between the ApoE isoforms, which is probably a limitation of the study. The levels of ApoE4 in plasma were under the limit of detection in our experiment. It is believed that brain ApoE does not cross to the periphery, but it may cross the BBB when it is bound to HDL (48). Indeed, the source of peripheral ApoE appears to be mainly the liver, with the brain and endocrine cells contributing little if any to plasma ApoE levels (48). In our experiment, we did not study the levels of HDL within the brain, as HDL in the periphery is mainly enriched with ApoAI, and its synthesis in glial cells is enriched with ApoE (48). In any case, ApoE appears not to play a fundamental role in the hypothesis of our study, although given the importance that the isoform ApoE4 plays in the context of alcohol and neuroinflammation, further studies are needed to ascertain possible implications of this specific isoform of ApoE.

This study highlights several events in the brain of male and female animals exposed to alcohol binge episodes. First, the specific binding of Lipid A to different apolipoproteins in the brains of males and females exposed to alcohol. Second, we detected different BECs achieved by males and females with the same doses of alcohol, with lower levels observed in females at the time of blood extraction. It is possible that the metabolism of alcohol is faster in females, as suggested elsewhere (75, 76, 77, 78), and the peak of BECs occurred at early times. Indeed, other markers are indicative of the activation of the acute-phase response in females, such as the plasma corticosterone levels, which was higher in females of the ethanol group. This higher corticosterone response in females than males after alcohol exposure was expected, according to the literature (79, 80, 81). Finally, as mentioned before, we found here a TLR4 upregulation in the PFC of males that underwent alcohol gavage administrations, which is in accordance with previous literature about the neuroinflammatory actions of ethanol in this area (13, 15, 61). In this study, the effect of ethanol on TLR4 upregulation in the PFC was not significant in females. Notably, disparities in the effects of alcohol in females versus males have been noted. Whereas some authors suggest that females may be more vulnerable to alcohol toxic effects than males (82, 83), other authors have shown that female rat brains appear to be more resistant to oxidative damage (84). Comparative studies showing the influence of ethanol in males and females using the same experimental approach at a time are still scarce. Biological differences between males and females and hormonal factors could interfere with the responses observed. For example, sex hormones in females may contribute to a possible neuroprotector effect, as has been suggested elsewhere (85, 86). In this study, the reproductive cycle was monitored in the females during experimentation, and no synchronization was observed among them. The lack of significance in TLR4 expression in alcohol-administered female rats in this study may reflect the protective effect of ApoAI in females, although we cannot discard that the peak of neuroinflammation in females was in an increasing/decreasing phase at the time-point when the sample was obtained (three hours after the last ethanol administration).

We are aware of the limitations of the study, some of which have also been described above: 1) Here, we reported several sexual differences regarding the expression of apolipoproteins, lipoproteins, BELs, corticosterone, etc. in plasma, but data from Western blot were uploaded and analyzed independently for each sex, which limits the total comparison between male and females in both alcohol and control conditions. In spite of this limitation of the study, we were able to identify a differential affinity for apolipoproteins to bind LPS components in male and female ethanol-treated animals, which constitutes an important result of this study. Altogether, these are undoubtedly novel results that open up doors in the field, but further studies are necessary to report results from a complete sexual difference perspective (ie differences in the expression levels in control animals, etc). 2) The choice of measuring Lipid A, among other parts of LPS, was based on its ability to interact with lipoproteins such as HDL through its Lipid A backbone (54). Studying how other parts of LPS, such as the Core, may interact with apolipoproteins is a goal for future studies. Additionally, in this study, we did not discern whether the binding of Lipid A to apolipoproteins takes place in the periphery, allowing the transport of LPS to the brain, or whether it takes place within the brain or in both compartments. It has been documented that LPS can alter BBB tight junction proteins and cross into the brain (87), although other authors have reported minimal penetration into the brain (30). 3) With this methodology, we cannot assure that the LPS components were able to cross the BBB. The pilot study showing decreases in LipidA in perfused animals but not in the aggregates suggest that the LipidA-apolipoprotein bound forms infiltrated in the brain, as demonstrated in our previous study with immunohistochemistry (31), but additionally, studies will be required to completely demonstrate it. Other studies have shown partial permeability of LPS to the brain parenchyma in different conditions and according to the dose (30) and by action of HDL or apolipoproteins (63, 88). Additionally, here we did not focus on the cellular specific localization of the aggregates and the response to this question needs of future and complementary immunohistochemical studies.

## Conclusion

In summary, our study shows the presence of small LPS components (Lipid A) bound to ApoAI in females and to ApoB in males in the PFC of animals exposed to alcohol intoxications, and no effects were found within the cerebellum. The impact of alcohol binges on TLR4 upregulation was observed in the PFC of alcohol-treated males, despite plasma LPS-binding protein was elevated in both sexes. Sexual differences were found in plasma ApoAI and ApoB expression, LDL, BECs, and corticosterone levels. The specific mechanism of the binding of LPS to different apolipoproteins, the cellular localization of the aggregates, and its functional consequences under alcohol intoxication conditions deserve further investigation to better understand the crosstalk between alcohol, neuroinflammation, and the neuroimmune response, as well as an exhaustive analysis from a sex/gender perspective.

## Ethics Statement

The animal study was reviewed and approved by the Animal Welfare Committee of Complutense University of Madrid (reference: PROEX 312/19) following European legislation (2010/63/EU).

## Data availability

All raw data generated in this study (such as complete Western blot images) are clearly classified, stored, and available to reasonable request to the corresponding author (lorio@psi.ucm.es).

## Supplemental data

This article contains supplemental data.

## Conflict of interest

The authors have no conflicts of interest to declare.
