# Supplementary material for: Bacterial lipopolysaccharide forms aggregates with apolipoproteins in male and female rat brains after ethanol binges
Source: J Lipid Res. 2024 Jan 29;65(3):100509. doi: 10.1016/j.jlr.2024.100509 (PMC10907226; doi:10.1016/j.jlr.2024.100509)
Supplement: Supplemental Data [file mmc1.docx]

**SUPPLEMENTARY INFORMATION**

1. **Supplementary Methods**

***Experimental design and binge ethanol treatment.***

Ethanol was administered intragastrically (3 g/Kg) every eight hours for four days, with an initial loading dose of 5g/Kg. To facilitate the collection once a day of vaginal smears, the female rats were divided into two groups. In one group, the estrous cycle was always analysed in the morning and in the other, always in the afternoon, according to the schedule below. Samples collection took place around 3 hours after the last ethanol i.g. administration.

**Table S1. Experimental design of the study**

| **TIME** | **DAY 1** | **DAY 2** | **DAY 3** | **DAY 4** | **DAY 5** |
| --- | --- | --- | --- | --- | --- |
| **7:00** |  | EtOH 3g/Kg | EtOH 3g/Kg | EtOH 3g/Kg | EtOH 3g/Kg |
| **10:00** |  |  |  |  | Samples´ collection |
| **11:30** | 1st group Vaginal smear | 1st group Vaginal smear | 1st group Vaginal smear | 1st group Vaginal smear |  |
| **15:00** | EtOH 5g/Kg | EtOH 3g/Kg | EtOH 3g/Kg | EtOH 3g/Kg |  |
| **18:00** | 2nd group Vaginal smear | 2nd group Vaginal smear | 2nd group Vaginal smear | 2nd group Vaginal smear |  |
| **23:00** | EtOH 3g/Kg | EtOH 3g/Kg | EtOH 3g/Kg | EtOH 3g/Kg |  |

***Estrous cycles in female rats***

**Fig. S1** **Schematic representation of unstained vaginal smear from females cycle phases**. The proportion of the three types of cells was used as a determinant of the cycle phases, finding epithelial cells (E), nucleated and round; cornified cells (C), without nucleus and irregular form; and leukocytes (L), round tiny ones. **A** Proestrus, with a predominance of epithelial cells. **B** Estrous, consisting mainly of cornified cells. **C** Metestrus, consists of the same proportion of the three types of cells. **D** Diestrus, with a predominance of leukocytes.

**Table S2**. **Specific antibodies used in western blotting to detect proteins of interest.**

| Protein | Primary Antibody | Secondary Antibody |
| --- | --- | --- |
| Ligands  ApoB  ApoAI  ApoE  Lipid A | 1:500 sc-393636  1:4000 Ab20453  1:500 sc-390925  1:500 Acris BP2235 | Mouse (1:2000)  Rabbit (1:3000)  Mouse (1:2000)  Goat (1:3000) |
| Receptors  LDLr  SRBI  TLR4  CD14 | 1:500 Ab30532  1:1000 NB400-104  1:500 BSA 1% sc-293072  1:1000 ab203294 | Rabbit (1:2000)  Rabbit (1:2000)  Mouse (1:2000)  Rabbit (1:2000) |
| Housekeeping  β-actin  GAPDH | 1:10000 A5441 Sigma  1:5000 G8795 Sigma | Mouse (1:10000)  Mouse (1:5000) |

**Table S3**. **Specific antibodies used in co-immunoprecipitation to precipitate the protein complex.**

| Co-IP Antibody | Primary WB Antibody | Secondary WB Antibody |
| --- | --- | --- |
| Lipid A  Acris BP2235 | ApoB 1:500 sc-393636  ApoAI 1:4000 Sc-135837  TLR4 1:500 BSA 1%  sc-293072 | Mouse (1:2000)  Rabbit (1:3000)  Mouse (1:2000) |
| TLR4 sc-293072 | Lipid A 1:500 Acris BP2235 | Goat (1:3000) |

1. **Supplementary Results**

**2.1 Determination of the estrous cycle phases in female rats**

Table S4 shows the estrous cycle of each female rat along the 4 days of experimentation. The distribution of phases in each animal was aleatory and no synchronization was observed.

**Table S4. Distribution of estrous cycle phases in female rats along experimentation**

**Fig. S2. Circle diagram showing the distribution of estrous cycle in the ethanol and control female animals.**

**
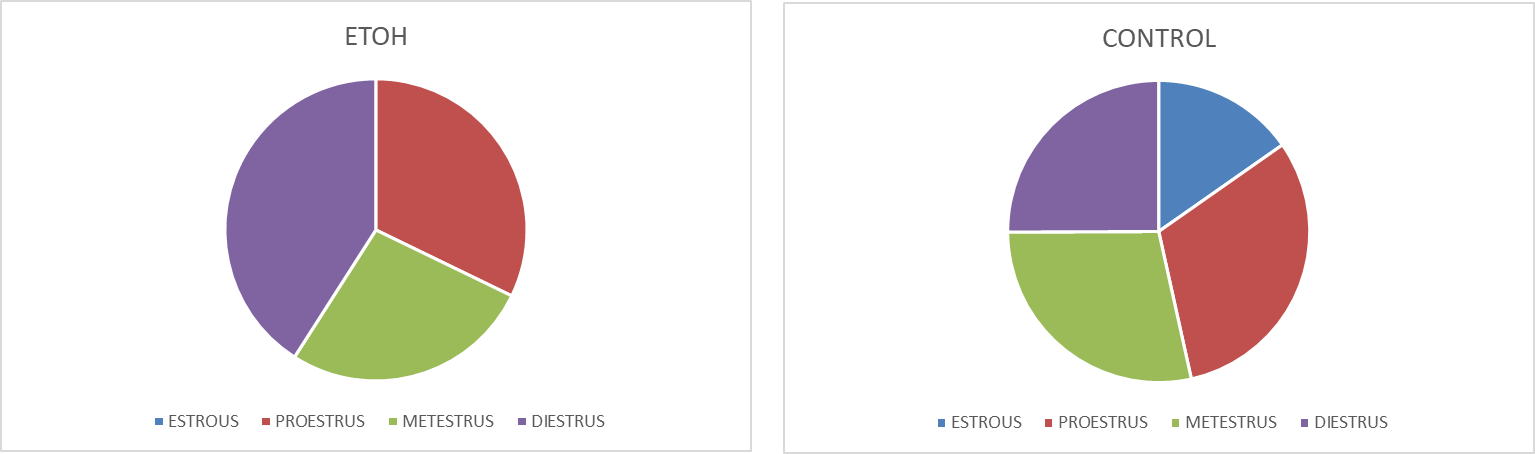
**

**2.2 Results in the cerebellum**

The whole study was repeated in the cerebellum, both in the vermis and in the cerebellar hemispheres. Next results show the data in the cerebellar hemispheres. Results in vermis showed no significant differences between groups (data not shown).

***Detection of LPS components (Lipid A and Core) and TLR4 in the cerebellar hemisphere of male and female alcohol intoxicated and control animals.***

In the cerebellar hemisphere, no significant differences were found in the Lipid A, Core and TLR4 levels between ethanol and control groups in males (Fig. S3 A,B,C; t_(16)_ =1.529, *p*=0.1457; t_(16)_ =1.410, *p*=0.1778; t_(16)_ =0.6408, *p*=0.5307, respectively),. Representative blots in Fig. S3D. In females, no changes in Lipid A, Core and TLR4 were found between ethanol and control groups (Fig. S3 E,F,G; t_(15)_ =1.958, *p*=0.0691; t_(15)_ =0.5886, *p*=0.5649; t_(15)_ =1.187, *p*=0.2538, respectively), although Lipid A levels in the female ethanol group showed a tendency to decrease (Student´s *t*-test near of significance, *p*=0.0691) that is contrary to the tendency observed in males. Representative blots in Fig. S3H.

**Fig. S3** **Detection of Lipid A and core and TLR4 expression in cerebellar hemisphere by western blotting**. The upper panel shows data in ethanol-treated (n=10) and control (n=8) males and the lower panel data in ethanol-treated (n=9) and control (n=8) females. **A** Expression of Lipid A in males. **B** Expression of the Core element of LPS in males. **C** Expression of TLR4 in males. **D** Representative blots. **E** Expression of Lipid A levels in females. **F** Expression of Core in females. **G** Expression of TLR4 levels in female rats. **H** Representative blots. Western blot data were normalized by β-actin and expressed as a percentage of change over controls. Results represent the mean ± S.E.M. of two technical replicates. No differences were observed between groups (trend in Lipid A in females (E): difference from control group *p*=0.0691; Student’s *t*-test).

***Detection of ApoAI, [LipidA-ApoAI] bound form and the receptor SRBI in the cerebellar hemisphere (Hcb) of male and female alcohol intoxicated and control animals.***

Regarding expression and binding to apolipoproteins in the cerebellar hemispheres, ApoAI was detected in this brain region but no differences were found in its total (Fig. S4A) or bound form (Fig. S4B) to Lipid A (t_(15)_ =0.5670, *p*=0.5791; t_(16)_ =0.7446, *p*=0.4673, respectively) or in the SR-BI expression (Fig. S4C, t_(16)_ =0.1125, *p*=0.9118) in males Hcb. Blots represented in Fig. S4D. Similarly, no differences were found in Lipid A, the [LipidA-ApoAI] bound form and the SR-BI expression in females (Fig. S4E, F & G; t_(15)_ =0.1165, *p*=0.9088; t_(15)_ =0.8680, *p*=0.3991; t(15)=1.012, *p*=0.3274, respectively). Representative blots in female animals shown in Fig.S4H.

**Fig. S4 Expression of ApoAI, [LipidA-ApoAI] bound form and SR-BI in cerebellar hemisphere**. The upper panel shows data in ethanol-treated (n=10) and control (n=8) males and the lower panel shows data in ethanol-treated (n=9) and control (n=8) females. **A** Expression of total ApoAI in males. **B** The ratio [LipidA-ApoAI] bound form in males, as indirect measurement of co-localization of proteins (bound form detected around 31 kDa and normalized by total ApoAI). **C** Expression of SR-BI in males. **D** Representative blots. **E** Expression of total ApoAI levels in females. **F** The ratio [LipidA-ApoAI] bound form in females, as indirect measurement of co-localization of proteins (Lipid A detected around 31 kDa and normalized by total ApoAI). **G** Expression of SR-BI levels in female rats. **H** Representative blots. Western blot data were normalized by β-actin and expressed as a percentage of change over controls. Results represent the mean ± S.E.M. of two technical replicates. No differences were observed between groups (Student’s *t*-test).

***Detection of ApoB, [LipidA-ApoB] bound form and LDLr in the cerebellar hemisphere of male and female alcohol intoxicated and control animals.***

Similarly, no differences were detected in total ApoB, [LipidA-ApoB] bound form or LDLr expression in males (Fig. S5A, B, C; t_(16)_=1.226, *p*=0.2378; Mann-Whitney U=38, *p*>0.05, n.s.; t_(16)_=1.822, *p*=0.0873, respectively; blots in Fig. S5D) or females (Fig. S5E, F, G; t_(15)_=1.049, *p*=0.3107; t_(15)_=0.4311, *p*=0.6725; t_(15)_=0.09363, *p*=0.9266, respectively; blots in Fig. S5H).

**Fig. S5 Expression of ApoB, [LipidA-ApoB] bound form and LDLr in cerebellar hemisphere**. The upper panel shows data in ethanol-treated (n=10) and control (n=8) males and the lower panel data in ethanol-treated (n=9) and control (n=8) females. **A** Expression of total ApoB in males. **B** The ratio [LipidA-ApoB] bound form in male Hcb, as indirect measurement of co-localization of proteins (Lipid A detected around 210 kDa and normalized by total ApoB). **C** Expression of LDLr in males. **D** Representative blots. **E** Expression of total ApoB levels in females. **F** The ratio [LipidA-ApoB] bound form in female Hcb, as indirect measurement of co-localization of proteins (Lipid A detected around 210 kDa and normalized by total ApoB). **G** Expression of LDLr levels in female rats. **H** Representative blots. Western blot data were normalized by β-actin and expressed as a percentage of change over controls. Results represent the mean ± S.E.M. of two technical replicates. No differences were observed between groups (Student’s *t*-test).

***Detection of ApoE, [LipidA-ApoE] bound form and ApoER2 in the cerebellar hemisphere of male and female alcohol intoxicated and control animals.***

ApoE was also detected in the cerebellar hemisphere in both groups with no significant differences in its total or bound form in males (Fig. S6A, B; t_(16)_ =1.517, *p*=0.1487; t_(16)_ =0.9982, *p*=0.3330, respectively). Interestingly, levels of ApoER2 in Hcb were upregulated in the ethanol group in males (Fig. S6C; Mann-Whitney U=10, *p*<0.05). Representative blots shown in Fig. S6D. Similarly, no differences were found in ApoE in its total or bound form in females (Fig. S6E, F; t_(14)_ =0.6143, *p*=0.5488; t_(13)_ =0.6697, *p*=0.5147, respectively). Contrary to what it was observed in males, ApoER2 was downregulated in the ethanol group (Fig. S6G; t_(14)_ =3.133, *p*=0.0073). Representative blots shown in Fig. S6H.

**Fig. S6 Expression of ApoE, [LipidA-ApoE] and ApoER2 in cerebellar hemisphere**. The upper panel shows data in ethanol-treated (n=10) and control (n=8) males and the lower panel data in ethanol-treated (n=9) and control (n=8) females. **A** Expression of total ApoE in males. **B** The ratio [LipidA-ApoE] bound form in male Hcb, as indirect measurement of co-localization of proteins (Lipid A detected around 36 kDa and normalized by total ApoE). **C** Expression of ApoER2 in males. **D** Representative blots. **E** Expression of total ApoE levels in females. **F** The ratio [LipidA-ApoE] bound form in female Hcb, as indirect measurement of co-localization of proteins (Lipid A detected around 36 kDa and normalized by total ApoE). **G** Expression of ApoER2 levels in female rats. **H** Representative blots. Western blot data were normalized by β-actin and expressed as a percentage of change over controls. Results represent the mean ± S.E.M. of two technical replicates. Different from control group: **p*<0.05, ***p*<0.01 (Mann-Whitney (S6C) or Student’s *t*-test (S6G)).
